# A qualitative exploration of the collaborative working between palliative care and geriatric medicine: Barriers and facilitators from a European perspective

**DOI:** 10.1186/s12904-016-0118-3

**Published:** 2016-05-11

**Authors:** Gwenda Albers, K. Froggatt, L. Van den Block, G. Gambassi, P. Vanden Berghe, S. Pautex, N. Van Den Noortgate, On behalf of EURO-IMPACT

**Affiliations:** Federation Palliative Care of Flanders (FPCF), Vilvoorde, Belgium; Department of Medicine, Division of Geriatrics and Gerontology, Ghent University Hospital, Ghent, Belgium; International Observatory on End of Life Care, Lancaster University, Lancaster, UK; End-of-life Care Research Group, Department of Family Medicine and Chronic Care, Vrije Universiteit Brussel (VUB) and Ghent University, Brussels, Belgium; Istituto di Medicina Interna e Geriatria, Università Cattolica Sacro Cuore, Rome, Italy; Division of Primary Care, University Hospitals Geneva, University of Geneva, Geneva, Switzerland

## Abstract

**Background:**

With an increasing number of people dying in old age, collaboration between palliative care and geriatric medicine is increasingly being advocated in order to promote better health and health care for the increasing number of older people. The aim of this study is to identify barriers and facilitators and good practice examples of collaboration and integration between palliative care and geriatric medicine from a European perspective.

**Methods:**

Four semi-structured group interviews were undertaken with 32 participants from 18 countries worldwide. Participants were both clinicians (geriatricians, GPs, palliative care specialists) and academic researchers. The interviews were transcribed and independent analyses performed by two researchers who then reached consensus.

**Results:**

Limited knowledge and understanding of what the other discipline offers, a lack of common practice and a lack of communication between disciplines and settings were considered as barriers for collaboration between palliative care and geriatric medicine. Multidisciplinary team working, integration, strong leadership and recognition of both disciplines as specialties were considered as facilitators of collaborative working. Whilst there are instances of close clinical working between disciplines, examples of strategic collaboration in education and policy were more limited.

**Conclusions:**

Improving knowledge about its principles and acquainting basic palliative care skills appears mandatory for geriatricians and other health care professionals. In addition, establishing more academic chairs is seen as a priority in order to develop more education and development at the intersection of palliative care and geriatric medicine.

**Electronic supplementary material:**

The online version of this article (doi:10.1186/s12904-016-0118-3) contains supplementary material, which is available to authorized users.

## Background

Populations are ageing in all European countries with a particularly rapid increase in the number of people over 80 years old [1]. Increasing life expectancy and the ageing of populations present significant challenges for the provision of optimal care to this population, particularly with respect to their needs for supportive and palliative care [2, 3].

Older people have a higher risk for developing multiple, chronic and progressive degenerative diseases [4]. In 2013, in the United Kingdom, 69 % of people aged 75 and over reported to have a long-standing illness [5]. Although, most older people will die from these chronic illnesses, the end of life is generally preceded by a lengthy period of decline and functional impairment [6]. In the course of illness, older people are at risk of receiving unnecessary medical investigations and treatments which are burdensome and expensive for the patient, family and society [7–10].

Several studies emphasize the importance of adopting palliative care approaches for non-malignant disease such as dementia [11, 12]. Recent studies in palliative care have also shown the potential benefits of an early palliative care approach [13–15]. The need to provide palliative care for all, regardless of diagnosis and prognosis and alongside potentially curative treatment can also be found in recent policy statements such as the World Health Organization (WHO) policy report advocating for palliative care as a component of integrated treatment throughout the life course [16].

In September 2012, the Maruzza Foundation, the European Association of Palliative Care (EAPC) and the European Union Geriatric Medicine Society (EUGMS) launched a joint manifesto ‘Palliative Care for Older People in the European Union’, at the European parliament, calling upon governments and institutions to ensure that every older citizen with chronic disease is offered the best possible palliative care approach wherever they are cared for [17].

Closer interaction between geriatric medicine and palliative care is of crucial importance in the promotion of high quality care for older people in the last years of their life. This has been recognized by the American Geriatrics Society and the American Academy of Hospice and Palliative Medicine who started a collaborative effort to convene medical leaders in both geriatric medicine and palliative care. They have identified overlapping interests and developed a joint proposal for ongoing collaboration in the areas of leadership and organizational structure, clinical care, education and training, public policy, and research [18]. However, the health care system in America differs from that in Europe where the organization and funding of health care and long-term care varies widely among countries.

A working group consisting of active members of the EUGMS and the EAPC was established in 2012. This European-level working group aimed to make recommendations on how to improve palliative care for older people in Europe that could be used by policy-makers and national organizations in order to better provide care for older people. A first step in reaching this goal was to identify existing collaborations and consider how future integration between palliative care and geriatric medicine in Europe could be nurtured.

Thus, a study was undertaken to answer the following research questions:

- What common ground exists between palliative care and geriatric medicine and what are the areas for possible collaboration?
- What are the barriers to closer collaboration between palliative care and geriatric medicine?
- What are the facilitators that (could) support collaboration and what are good practice examples of collaboration/integration between palliative care and geriatric medicine?

## Methods

### Design

This descriptive exploratory study, located in an interpretivist frame, used semi-structured group interviews as the data collection method [19]. This qualitative approach was chosen because it allows for exploratory discussion and interaction to explore the range of views and experiences of participants.

### Setting and participants

Participants were recruited from the two main discipline annual conferences (EUGMS congress in October 2013 and EAPC congress in June 2014). An invitation email was sent by members of the EUGMS working group to all people who were registered for the 2013 EUGMS congress and by the EAPC secretary to all those registered to participate in the 2014 EAPC congress. Those people who replied first to inform us that they were interested were mostly accepted to participate. Participation was limited to a maximum of 12 people per group. Those accepted to participate were divided over two groups at both congresses in order to have four groups with gender and age heterogeneity, variable professional experience and from different countries.

### Data collection

A semi-structured interview guide consisting of open questions and a set of prompts for each question was developed and reviewed within the multidisciplinary research team (Additional file 1). The following themes were covered (1) barriers to collaboration; (2) facilitators for collaboration; (3) initiatives that were successful in promoting or improving collaboration and factors that made these initiatives successful or failing. These dimensions were considered under four main areas: clinical care, education and training, policy, leadership and organizational structures, drawn from domains described in the report of the Geriatrics – Hospice and Palliative Medicine Work Group [18].

The group interviews took place in a separate room at the congress venue. An experienced moderator (KF) led the group interviews and two observers (GA and NVdN or SP) took notes. All interviews were conducted in English and audiotaped. Each interview took about 60 minutes. Informed signed consent was obtained from participants and all participants completed a short demographic questionnaire.

### Data analyses

The interviews were transcribed. Independent analyses were performed by two researchers (GA and KF), using a coding template based on the interview schedule [20]. Barriers and facilitators were extracted from the initiatives that were described as successful or failing in promoting or improving collaboration between palliative care and geriatric medicine. When GA and KF reached consensus on the classification of the barriers and facilitators the other authors were given the opportunity to comment on the categorization and clarity of the classification. After two rounds of written feedback all authors agreed on the classification of the barriers and facilitators and how they are described in the results section.

### Ethical considerations

The Medical Ethics Commission of the Brussels University Hospital was consulted. They concluded no ethical approval was needed for this study because no active involvement of patients or patient information was involved. Signed consent for use of data from the group interviews for analysis and dissemination was obtained from all participants.

## Results

A total of 32 participants (22 women and 11 men) from 18 countries worldwide (Australia, Austria, Belgium, Denmark, England, Finland, Germany, Italy, Ireland, Israel, Netherlands, New Zealand, Norway, Poland, Portugal, Spain, Switzerland, USA) took part in four group interviews. Participant characteristics are presented in Table 1.

**Table 1:** Characteristics of participants (*n* = 32)

Characteristics	Interview 1	Interview 2	Interview 3	Interview 4	Total
	(*n* = 8)	(*n* = 7)	(*n* = 8)	(*n* = 10)	(*n* = 32)^a^
Gender					
Male	3	3	4	1	11
Female	5	4	4	9	22
Age (years)					
≤29	1	0	0	1	2
30–39	1	3	1	2	7
40–49	0	0	0	2	2
50–59	4	2	4	5	15
≥60	2	2	2	0	6
Clinical work experience (years)					
1–9	1	1	1	3	6
10–19	3	2	5	4	14
20–29	1	3	1	3	8
≥30	3	1	1	0	5
Work setting^b^					
Hospital	3	5	0	0	8
Academic hospital	5	2	2	3	12
Long-term care facility	0	1	3	2	6
Hospice/palliative care unit	0	0	3	2	5
University/other	0	0	0	5	5
Position^c^					
GP	0	0	3	2	5
Geriatrician	7	7	2	1	17
Palliative care specialist	0	1	3	3	7
Internist	1	0	0	0	1
Nurse	0	0	1	2	3
Bereavement coordinator	0	0	1	0	1
Policy adviser	0	0	1	0	1
Researcher	1	0	1	6	8

^a^ One of the participants of group interview 3 also participated in group interview 1

^b^ Some participants work in more than one setting or have more than one position

^c^ Some participants hold more than one position (most palliative care specialists are also GP or geriatrician)

### Common ground and areas for possible collaboration between palliative care and geriatric medicine

A number of areas of synergy, similarities or areas of overlap between palliative care and geriatric medicine with respect to clinical care, were identified by participants (Table 2).

**Table 2:** Common ground in palliative care and geriatric medicine

	Palliative care →	*Common ground*	← Geriatric medicine
tructure/process Indicators^a^	(Knowledge of) basic palliative care	*Patient population (cannot be cured, last stage of life)*	Geriatric assessment
	Ethical decision-making	*Holistic approach*	Importance of rehabilitation for dignity feeling
	Prognostication	*Integration of care, providing good care*	
	Goal setting	*Advance care planning*	
Outcome Indicators^a^		*Quality of life*	Frailty and functional status
		*Comfort*	Co-morbidity
		*Dementia*	Metabolism of the older patient
		*Delirium*	Pharmacology

^a^ The areas of differences and commonalities are categorized into structure and process, and outcome indicators in accordance with Donabedian’s health system analysis approach [25]. An indicator refers to a measurable element of practice or system which could indicate what can be a priority to improve quality of care, or in this case, what can be done to bring together palliative care and geriatric medicine. ‘Structure’ refers to the attributes of the settings in which care occurs, includes the attributes of material and human resources and of organizational structure. ‘Process’ refers to what is actually done in giving and receiving care. ‘Outcome’ measures attempt to describe the effects of care on the health status of patients and populations

Table 2 presents the areas of difference and commonality categorized into structure and process indicators and outcome indicators. Clinically, the patient population of interest was recognised as very similar:

*… older people often have diseases that cannot be cured anymore. So in that sense they [older people]-- when you look at the definition of palliative care they belong to-- they have the right to that care [palliative care]. (PC medical specialist and elderly care physician, NL, I3)*

It was indicated that dealing with a similar patient population, sharing similar approaches to care and common goals provide possibilities for collaborations between palliative care and geriatric medicine. Participants from each discipline also reported what would be the respective value that would emerge from collaboration. It was reported that palliative care would bring knowledge of general palliative care, support in ethical decision-making, prognostication and goal setting. Geriatric medicine, instead, would contribute knowledge of geriatric assessments that leads to better a understanding of frailty and functional status, comorbidity, metabolism, and pharmacology and also the importance of rehabilitation and physiotherapy.

### Barriers and facilitators for collaboration between palliative care and geriatric medicine

Table 3 presents an overview of the barriers and facilitators for collaboration between palliative care and geriatric medicine.

**Table 3:** Barriers and facilitators for collaboration between palliative care and geriatric medicine

	Barriers	Facilitators
Clinical practice	- Lack of understanding and knowledge of the other discipline	- Cross-disciplinary work, e.g. inter-professional teams, multi-disciplinary team working, consultation/expert advice from the other discipline
	- Disciplinary identity	- Advance care planning
	- Lack of communication between disciplines and settings	- The role of the GP providing generalist palliative care in the community setting
Education and training	- Lack of educational opportunities on palliative care or geriatric medicine within the other disciplines, and lack of shared trainings between the disciplines	- A mandatory internship within the other discipline
		- Palliative care and geriatric medicine perspectives are presented at each other’s conferences
Strategic/policy level	- Non-existence of palliative care and/or geriatric medicine as specialty	- Defining core competences in palliative care for geriatricians and other health care professionals
	- Small number of academic chairs in both palliative care and geriatric medicine	- Strong leadership
	- Organization and financing of health care	- Establishing taskforces, interest groups lobbying and working around themes that benefit both disciplines

#### Barriers for collaboration between palliative care and geriatric medicine

A number of barriers were identified that hindered collaborative working between palliative care and geriatric medicine with respect to clinical practice. These concerned knowledge about each other’s disciplines, disciplinary identity and communication.

Participants indicated that one barrier to collaboration was a lack of shared understanding and knowledge of what palliative care and geriatric medicine respectively offer:

*One of the challenges I can see is that still some people reduce palliative care to symptom management and not holistic, the real holistic way, and I think of social and psychological issues (Geriatrician, Norway, I1)*

Participants articulated the different focus of each discipline:

*In practice the big difference for us is the aim of our intervention because in palliative care we are very concerned with comfort. I can say that comfort is our primary outcome. And in geriatric care our outcome is the autonomy of the patient and the functionality. I think that is one of the biggest differences. (Nurse/researcher, Portugal)*

These differences would have implications for the care being provided. For example, in some countries [Italy, Spain, Denmark, Belgium and the UK], palliative care was described by participants as being considered mostly for older patients with cancer. Consequently, there is often a lack of recognition of the palliative care needs of people with conditions other than cancer.

*The palliative care doesn’t look at these patients. They have mostly cancer patients still. (Male general practitioner (GP), Denmark, I3)*

*The difficulty is the move from being under a geriatrician to moving into specialist palliative care. And actually very few older people unless they have oncology will be seen by a specialist palliative care. (Researcher, UK, I4)*

Several participants brought up tensions that exist between palliative care and geriatric medicine, reflecting the difficulties that occur when disciplines expand or change their focus of interest. Among participants from the geriatric field, a move to palliative care was understood as a providing terminal care, focusing only on those about to die:

*We have now two groups of geriatricians, some are very familiar with palliative care and the other groups who have the fear that if we talk too much about palliative care in geriatrics that it becomes like ‘OK that is the program for old people, palliative care, no curative interventions and we save money’. (Geriatrician, Switzerland, I2)*

Another perspective was that as geriatric medicine expands its knowledge and practice this might reduce the need for a specialist palliative care approach:

*From a geriatrician’s perspective I think the palliative care doctors or organizations they are afraid that they are not necessary anymore because the geriatrician sees everything, we have oncology, we have pain management, we have dementia, heart failure, COPD, we have all this. The palliative care only looks at the small part, the last few weeks or months, or half year. So that is the reason that they are afraid that they [Geriatricians] can do it by their own. (Geriatrician, Germany, I1)*

On a more practical level it was reported that a lack of communication between disciplines and settings hinder the provision of collaborative care:

*I think it’s an important issue that what we find obvious as being good palliative care mainly multi-disciplinary work. Really coming together, take decisions together with nurses, doctors. It’s less obvious in-- at home with a geriatric patient for instance. Everyone does his work. There’s a lack of communication and goals aren’t discussed about, aren’t documented and that’s a problem. (GP and PC specialist, Belgium, I3)*

Barriers concerning education and training were shared issues in palliative care and geriatric medicine:

*I think an extra challenge these disciplines have is recruitment. Geriatrics and palliative medicine neither is very popular among medical students. So I think we share the common problems, bringing talented students (Geriatrician, Norway, I1).*

Collaboration was hindered owing to a lack of educational opportunities within disciplines, but also shared training between the disciplines.

*In Finland we have these 2 courses, the other one is privately funded, 5 years every time, but we don’t have university education for palliative medicine. (Geriatrician, Finland, I2)*

In addition, a number of barriers were identified at the higher, national policy level. It was reported that in some countries palliative care or geriatric medicine are not recognized as a specialty. It was proposed by participants that the low status of the disciplines is reflected by small numbers of academic chairs in both palliative care and geriatric medicine.

*Concerning Portugal there is a geriatric chair at some universities, not at all [universities] and concerning palliative care only optional courses during the pre-graduation, but no obligatory ones. And during the course for geriatrics there is one lecture of palliative care. (Geriatrician, Portugal, I1)*

Policy around the organization and financing of health care also influences the possibilities for collaboration and the provision of good care for older people.

*The problem is the fragmentation, in Spain we have 17 different health care systems. In Madrid region there is an official strategy for palliative care with the head of the department of health and that settled reacts with a palliative care units of all Madrid hospitals and also home care palliative units. So there is a strategy that covers 100 %, both home and hospitals but it is only Madrid, you move a few kilometers and you don’t get that. (Geriatrician, Spain, I1)*

#### Facilitators for collaboration between palliative care and geriatric medicine

Facilitators for collaboration between palliative care and geriatric medicine in clinical care, were identified among those supported by examples focused upon the different ways in which cross-disciplinary work could be supported. Participants talked about the integration of care since both disciplines sought to achieve this in their daily practice:

*.. palliative care is about integration. Gerontology is about integration. And both of them involve inter-disciplinary team work. And it’s all about integration really. (GP, Ireland, I4)*

Inter-professional teams were also indicated as a source of expert advice and consultation, around ethical issues, clinical management, decision making among people with multiple chronic conditions:

*I mean when you have a difficult decision for feeding, maintaining treatment, sometimes just to have someone with expertise in ethical decisions and to have the opportunity to discuss, that is really helpful for me, a strong support. (Geriatrician, Belgium, I2)*

*So the oncologist approach us because they see that their patients more and more are heavily, multimorbid and they have no clue at all how to do decision-making for therapies. So they ask us to help them with assessment instruments. (Geriatrician, Austria, I1)*

Integrated care was also supported by what some participants termed as multi-disciplinary team working:

*So the team approach in geriatrics and in palliative care is for me essential. (GP and palliative care specialist, Israel, I4)*

Examples of multidisciplinary teams were given:

*In my hospital it is a geriatrician, an internist and a family doctor and oncologist in the palliative care team. (Geriatrician, Spain, I1)*

Multidisciplinary team working was also described as providing opportunities for collaborative, mutual, learning:

*We have an interest group in the hospital where I work and this is a multidisciplinary group of nurses and doctors and there is one physiotherapist as well between palliative department and geriatric department. And this has led to collaboration between the nurses, they go to visit each other and give teaching about geriatric issues and on the other hand the palliative care nurses about palliative care issues. (Geriatrician, Germany, I1)*

The role of the GP was reported as an important bridge between palliative care and geriatric medicine. In relation to this, the setting where care was being provided was reported as a barrier, as geriatricians were more often affiliated to acute hospitals than community settings.

*… in many cases it is the GP who visits a very old patient at home or in a nursing home. They have to know what is palliative care and geriatric medicine. I think it is very important that a geriatrician can intervene when it is necessary but the core business is for GPs I think of geriatric medicine. (Geriatrician, Austria, I1)*

Already referred to earlier, the process of decision-making around care, was an area where consultation across the disciplines could ensure better care for older people. Related to this was the process of advance care planning that was identified as a process that could bring together palliative care and geriatric medicine.

*If you ask me to point out one thing we can do here and now, I think, it’s much more early meeting with the family. As it is we call them a coordinating meeting with the family, with the home nurse, to plan. Advance care planning at an early stage. I think that solves so much. Giving each other the mobile number. Who to contact when and all these things. I think that’s very important. (GP and PC medical specialist, Belgium, I3)*

Facilitators were identified at a more strategic or policy level.

Palliative care and geriatric medicine should be recognized as medical specialties to further flourish and enable collaboration.

*Each specialty that is a recognized specialty has a section and sits in Brussels EUMS [European Union of Medical Specialists]. Maybe that is something that should be done because there are countries in Europe that already have palliative medicine specialties or subspecialties. And that is how the geriatrics group is also working. One of its aims is to promote the specialty in countries where they don’t have the specialty. And doing it with a sort of European idea, like having the European lobbying behind. (Geriatrician, Austria, I2)*

It was also reported that defining core competences in palliative care for geriatricians and for health care professionals in general would be useful to integrate palliative care into geriatric training.

*I was wondering whether we could define core competencies in palliative care together in the [geriatric] syllabus. That should be included anyway in the syllabus. In the model of integrated care this has to be part of pre- and post-graduation training and of course this would fit in in the role of professionalism/leadership, as well as a fourth column in a training in other specialties. (Geriatrician, Germany, I1)*

In addition, it was mentioned that a mandatory internship in palliative care for geriatricians in training could provide an adequate learning opportunity.

*So if you do your geriatric training, I think you can do a placement in palliative care. And if you do a palliative care you can do a placement in geriatrics. But it’s not mandatory. So that’s what you’re saying it should be. Part of the curriculum as opposed to a ‘You can do it if you want to do it’. (Researcher, Australia, I4)*

More informal opportunities for mutual learning were also mentioned, for example, ensuring palliative care and geriatric medicine perspectives are presented at each other’s conferences.

All this educational effort was to be supported by strong leadership.

*It is very individual, it depends on the attitude of a single person in the hospital, at the surgery department or at the emergency department or geriatric department. It depends on this individual attitude. (Geriatrician, Austria, I1)*

Also at strategic/policy level it was reported that strong leadership is important in order to establish task forces or interest groups lobbying and working around a specific theme that benefits both disciplines.

*We from the geriatric society made a special interest group and the German society of palliative medicine which was very open to accept which is not always the case in a society where you want to get in so it was an easy way in a way to get in there (Geriatrician, Germany, I1)*

## Discussion

This study has explored current experiences and perspectives about the collaborations between palliative care and geriatric medicine. To our knowledge this is the first European study exploring barriers and facilitators for collaboration at different levels, namely at clinical practice, education and training level and a strategic/policy level. We found that limited knowledge of what the other discipline offers, a lack of common practice and limited communication between disciplines and settings were considered as barriers for collaboration. At the level of clinical practice several initiatives such as multi-disciplinary team work and consultation were recognized as able to promote the integration. In addition, it was considered that the recognition of palliative care and geriatric medicine as specialties and the development of accompanying competencies and curricula to support them, alongside strong leadership would help to enable collaboration.

Participants reported that in some cases care is being provided that meets palliative principles but which is not called or labeled as palliative care. However, it was also identified that palliative care is still perceived by many health care professionals, including geriatricians, as only being relevant to cancer patients and those in the very last phase of life. It might be that when geriatricians think of palliative care they think about specialist palliative care services or teams whereas this type of service is intended to only manage the more complex and difficult cases [3]. At the same time, it was mentioned that referral to specialist palliative care was difficult because of a priority being given to cancer patients in many countries. Improving knowledge about palliative care among geriatricians and other health care professionals seems imperative. A model that might be the basis of such a development has been proposed by Quill and Abernethy [21]. They argue that basic general palliative care skills need to be incorporated into every medical specialty’s practice (general palliative care) [21]. These general palliative care skills include: basic pain and symptom management, treatment of general depression and anxiety, and ability to facilitate discussion about prognosis, goals of treatment, suffering, and code status. This is in line with the palliative care approach defined by the Council of Europe [22] and WHO [16].

Given the overlap in the population served by palliative care and geriatric medicine, a clear opportunity for collaboration and shared learning activities exist. Both palliative care and geriatric medicine have a multidisciplinary and holistic approach, and they both strive to improve quality of life of people with serious chronic illnesses. Consequently, both palliative care and geriatric specialists could benefit from common learning activities around issues such as symptom control, communication, decision-making in challenging ethical situations such as dementia, multi-dimensional assessment, frailty syndrome and co-morbidity.

In most European countries such teaching activities have increased in the last years. However, there remains a need for reinforcing and harmonizing those activities to prepare future health care professionals face the projected increase in chronic and disabled older people. The lack of shared training programs might be explained by the small number of academic chairs in both palliative care and geriatric medicine which are important for raising the profile of specialties within clinical and academic arenas. The EAPC Atlas Palliative Care in Europe 2013 [23] showed that in 14 out of 30 European countries there have been full professors in palliative medicine in faculties of Medicine. In 25 countries palliative medicine is taught as a subject (mandatory or optional) at medical schools, in 17 countries palliative care is not taught as a course or subject at medical schools and for 11 countries there was no information available. There is a similar pattern in geriatric medicine. A Europe-wide study showed that geriatrics is a recognized medical specialty in 16 and a subspecialty in 9 out of 33 European countries surveyed [24].

Strong leadership was identified as a facilitator both at a clinical practice level as well as at a strategic/policy level. Key individuals are needed to establish task forces and interest groups working on palliative care within a geriatric society and vice versa. Strong leadership at local and national level supports personal liaising, lobbying and ongoing communication between organizations’ members and development of new initiatives at the intersection of the two disciplines.

The organization and/or fragmentation of health care, the way care is financed and the fact that older people are cared for by various care professionals over a variety of settings complicates and seems to hinder a possible collaboration between palliative care and geriatric medicine. The provision of specific palliative care services varies greatly over European countries. Hospital palliative care support teams and home care teams who provide advice and support to other clinical staff are supposed to work in close collaboration with other specialists. As such, palliative care support teams could support geriatricians in providing the best possible care for older people. Conversely, geriatricians can support palliative care specialists in complex situations. Correspondingly, we recommend action research on interdisciplinary teamwork and leadership in palliative care for geriatric patients. It would also be helpful to understand the patient and family perspective and a perspective of service users and get them involved in future research on this topic.

The exploratory study design and the compositions of participants in the group interviews, of whom many were geriatricians with a particular interest for palliative care, may be seen as a limitation. However, because of their motivation to participate we were able to provide insights contributing to a better understanding of collaboration between the two disciplines and how collaboration could be promoted. Moreover, the aim of the current study is in line with one of the key recommendations of the collaborative effort of the American Geriatrics Society and the American Academy of Hospice and Palliative Medicine, to identify areas of resistance to collaboration [18].

## Conclusion

Considering the growing need of palliative care for older people, improving knowledge about palliative care principles and acquainting general palliative care skills of geriatricians and other health care professionals is of crucial importance. However, whilst there are good examples of close clinical working between the disciplines, e.g. multidisciplinary team working, there is very limited collaboration in education and policy. Limited understanding about what the other discipline offers, a lack of common practice and limited communication between disciplines and settings were considered as barriers for collaboration between palliative care and geriatric medicine. To this end, establishing more academic chairs is seen as a priority and would be an important facilitator for further education and development at the intersection of the two disciplines. This could also result in a better collaboration between and integration of palliative care and geriatric medicine.

### Availability of data and materials

The datasets supporting the conclusions of this article are available from the corresponding author upon request.

## Additional file

Additional file 1: Interview Guide. (DOCX 16 kb)

## Abbreviations

EAPC: European association of palliative care
EUGMS: European union geriatric medicine society
GP: General practitioner
WHO: World health organization

## References

[1] United Nations. World Population Ageing. (2013). Available at: http://www.un.org/en/development/desa/population/publications/pdf/ageing/WorldPopulationAgeing2013.pdf (Accessed 22 April 2016).

[2] Hall S, Petkova H, Tsouros AD, Costantini M, Higginson IJ, World Health Organisation (WHO) (2011). Palliative Care for Older People: better practices. Palliative Care for Older People: better practices.

[3] Van den Block L, Albers G, Pereira S, Onwuteaka-Philipsen B, Pasman R, Deliens L (2015). Palliative care for older people : a public health perspective.

[4] Matzo ML (2004). Palliative Care: prognostication and the chronically ill: methods you need to know as chronic disease progresses in older adults. Am J Nurs.

[5] Adult Health in Great Britain, 2013. Office for National Statistics Available at: http://www.ons.gov.uk/ons/dcp171778_398686.pdf (Accessed 22 April 2016).

[6] Evers MM, Meier DE, Morrison RS (2002). Assessing differences in care needs and service utilization in geriatric palliative care patients. J Pain Symptom Manage.

[7] Pautex S, Curiale V, Pfisterer M, Rexach L, Ribbe M, Van Den Noortgate N (2010). A common definition of geriatric palliative medicine. J Am Geriatr Soc.

[8] Piers RD, Benoit DD, Schrauwen WJ, Van Den Noortgate NJ (2010). Factors influencing ICU referral at the end of life in the elderly. Z Gerontol Geriatr.

[9] Sampson EL, Gould V, Lee D, Blanchard MR (2006). Differences in care received by patients with and without dementia who died during acute hospital admission: a retrospective case note study. Age Ageing.

[10] Ryan T, Gardiner C, Bellamy G, Gott M, Ingleton C (2012). Barriers and facilitators to the receipt of palliative care for people with dementia: the views of medical and nursing staff. Palliat Med.

[11] Birch D, Draper J (2008). A critical literature review exploring the challenges of delivering effective palliative care to older people with dementia. J Clin Nurs.

[12] van der Steen JT, Radbruch L, Hertogh CM, de Boer ME, Hughes JC, Larkin P (2014). White paper defining optimal palliative care in older people with dementia: a Delphi study and recommendations from the European Association for Palliative Care. Palliat Med.

[13] Temel JS, Greer JA, Muzikansky A, Gallagher ER, Admane S, Jackson VA (2010). Early palliative care for patients with metastatic non-small-cell lung cancer. N Engl J Med.

[14] Hall S, Kolliakou A, Petkova H, Froggatt K, Higginson IJ (2011). Interventions for improving palliative care for older people living in nursing care homes. Cochrane Database Syst Rev.

[15] Morrison RS, Dietrich J, Ladwig S, Quill T, Sacco J, Tangeman J (2011). Palliative care consultation teams cut hospital costs for Medicaid beneficiaries. Health Aff (Millwood).

[16] World Health Organization. Strengthening of palliative care as a component of integrated treatmentthroughout the life course. EB134/28. 134th session; 2013.

[17] European Association for Palliative Care, European Union Geriatric Medicine Society. Better Palliative Care for Older People: Joint Manifesto. Available at: <http://www.eapcnet.eu/Themes/Specificgroups/Olderpeople/EuropeanParliamentMeeting.aspx> (Accessed August 2015).

[18] Report of the Geriatrics-Hospice and Palliative Medicine Work Group (2012). American Geriatrics Society and American Academy of Hospice and Palliative Medicine leadership collaboration. J Am Geriatr Soc.

[19] Frey JH (1991). The group interview in social research. Soc Sci J.

[20] Crabtree BF, Miller WL, Crabtree BF, Miller WL (1999). Using codes and code manuals: a template organizing style of interpretation. Doing Qualitative Research.

[21] Quill TE, Abernethy AP (2013). Generalist plus specialist palliative care--creating a more sustainable model. N Engl J Med.

[22] Council of Europe (2003) Recommendation Rec (2003) 24 of the Committee of Ministers to memberstates on the organisation of palliative care.

[23] Centeno CLT, Donea O, Rocafort J, Clark D (2013). EAPC Atlas of Palliative Care in Europe.

[24] Michel JP, Huber P, Cruz-Jentoft AJ (2008). Europe-wide survey of teaching in geriatric medicine. J Am Geriatr Soc.

[25] Donabedian A (1980). The Definition of Quality and Approaches to Its Assessment. Vol 1. Explorations in Quality Assessment and Monitoring.

