# Supplementary material for: A qualitative exploration of the collaborative working between palliative care and geriatric medicine: Barriers and facilitators from a European perspective
Source: BMC Palliat Care. 2016 May 11;15:47. doi: 10.1186/s12904-016-0118-3 (PMC4866297; doi:10.1186/s12904-016-0118-3)
Supplement: Additional file 1: — Interview Guide. (DOCX 16 kb) [file 12904_2016_118_MOESM1_ESM.docx]

**INTERVIEW GUIDE DISCUSSION GROUPS**

1. **Tasks and skills**

- What are the **main tasks of palliative care specialists / geriatricians** in providing optimal care to older people with chronic diseases in their last years of life?
- What are the **main palliative care skills that geriatricians need** to be trained in to be able to offer an optimal palliative care approach to older people with chronic diseases in their last years of life?
- What **skills can palliative care specialists / geriatricians add to the other field** to provide optimal care to older people with chronic diseases in their last years of life?

1. **Collaboration between palliative medicine/care and geriatric medicine**

- How could collaboration occur between palliative medicine/care and geriatric medicine? Think for example about…:

1. Clinical care (practice level in own country)
2. Education and training (national and European level)
3. Research (national and European level)
4. Policy (national and European level)
5. Leadership and organizational structures (national and European level)

- Can you think of any initiatives that were successful in promoting or improving collaboration? In your country or on a European level?
- What made these initiatives successful?

1. **Barriers to collaboration between geriatric medicine and palliative medicine/care**

- What barriers are there for collaboration between geriatric medicine and palliative care?
- If you think for example about…:

1. Clinical care (practice level in own country)
2. Education and training (national and European level)
3. Research (national and European level)
4. Policy (national and European level)
5. Leadership and organizational structures (national and European level)

- How can these barriers overcome?
- Can you think of any initiatives that failed in promoting or improving collaboration? In your country or on a European level?
- What happened that led to these initiatives not succeeding?
